# Modeling Structured Dependency Tree with Graph Convolutional Networks for Aspect-Level Sentiment Classification

**DOI:** 10.3390/s24020418

**Published:** 2024-01-10

**Authors:** Qin Zhao, Fuli Yang, Dongdong An, Jie Lian

**Affiliations:** 1 Department of Computer Science and Technology, Shanghai Normal University, Shanghai 200234, China; q_zhao@shnu.edu.cn (Q.Z.); 1000512717@smail.shnu.edu.cn (F.Y.); andongdong@shnu.edu.cn (D.A.); 2 Key Laboratory of Embedded Systems and Service Computing of Ministry of Education, Tongji University, Shanghai 201804, China

**Keywords:** sentiment analysis, aspect sentiment analysis, graph neural network, structured dependency tree

## Abstract

Aspect-based sentiment analysis is a fine-grained task where the key goal is to predict sentiment polarities of one or more aspects in a given sentence. Currently, graph neural network models built upon dependency trees are widely employed for aspect-based sentiment analysis tasks. However, most existing models still contain a large amount of noisy nodes that cannot precisely capture the contextual relationships between specific aspects. Meanwhile, most studies do not consider the connections between nodes without direct dependency edges but play critical roles in determining the sentiment polarity of an aspect. To address the aforementioned limitations, we propose a Structured Dependency Tree-based Graph Convolutional Network (SDTGCN) model. Specifically, we explore construction of a structured syntactic dependency graph by incorporating positional information, sentiment commonsense knowledge, part-of-speech tags, syntactic dependency distances, etc., to assign arbitrary edge weights between nodes. This enhances the connections between aspect nodes and pivotal words while weakening irrelevant node links, enabling the model to sufficiently express sentiment dependencies between specific aspects and contextual information. We utilize part-of-speech tags and dependency distances to discover relationships between pivotal nodes without direct dependencies. Finally, we aggregate node information by fully considering their importance to obtain precise aspect representations. Experimental results on five publicly available datasets demonstrate the superiority of our proposed model over state-of-the-art approaches; furthermore, the accuracy and F1-score show a significant improvement on the majority of datasets, with increases of 0.74, 0.37, 0.65, and 0.79, 0.75, 1.17, respectively. This series of enhancements highlights the effective progress made by the STDGCN model in enhancing sentiment classification performance.

## 1. Introduction

With the exponential growth of information on the Internet, extracting public opinion tendencies has become an important task. To achieve this, sentiment analysis of the information is necessary. Aspect-Based Sentiment Analysis (ABSA) is a fine-grained task in sentiment analysis that aims to predict sentiment polarities (e.g., positive, neutral, negative) of specific aspects in a sentence. Compared to document-level and sentence-level sentiment analysis, aspect-based analysis can more precisely extract sentiment polarities of specific aspects in a sentence, thus better capturing user viewpoints. For example, for review “*The food is extremely tasty, but the service is dreadful*”, opposite sentiment polarities are expressed for the two aspects, “*food*” and “*service*” in the sentence: a positive sentiment is expressed towards the food, while a negative sentiment is expressed towards the service.

The key to aspect-based sentiment analysis lies in extracting relationships between aspect and corresponding opinion words to infer the sentiment polarity of the aspect. Traditional aspect-based sentiment analysis [1] utilizes sentiment lexicons to assign different sentiment polarities or scores to opinionated words, and conducts aggregation based on the assigned weights to obtain overall sentiment tendencies. However, this approach requires constructing and maintaining sentiment lexicons, which poses great challenges. Some machine learning-based aspect sentiment analysis [2] uses hand-crafted features to train classifiers, which demands extensive human efforts. In recent years, with the development of deep learning, neural network models have received increasing attention from researchers and found wide applications. Earlier studies [3] adopted RNN models to capture sequential information in sentences for extracting semantic relationships between aspects and opinion words. However, they suffered from problems like semantic information loss and vanishing gradients. To address these limitations, LSTM networks [4,5] have been widely employed for aspect-level sentiment analysis tasks and achieved better effects in modeling long sequence data and capturing long-term dependencies. Recently, with the advancement of graph neural networks, applying GCN and GAT to sentiment analysis has become a hot topic receiving extensive attention.

**Figure 1:**
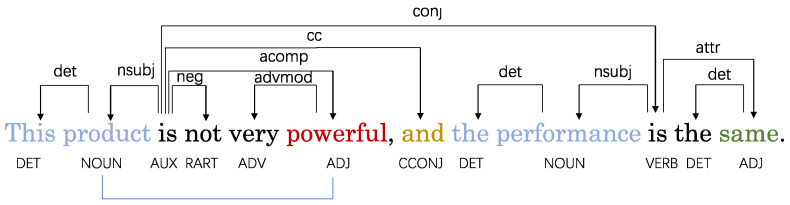
An example to illustrate the usefulness of part-of-speech dependence. The dependencies can be inferred by some key words in the sentence; we can easily guess that there is a strong dependence between “*product*” and “*powerful*”.

Some researchers construct dependency graphs using dependency parsers and leverage graph neural networks to obtain neighbor node information in order to utilize syntactic knowledge of the sentences for capturing relationships between aspects and contextual information [6,7]. However, there are still some limitations with the current dependency graphs. First, in the dependency parse tree, the edge weights between nodes are binary, regarding nodes with dependencies as equally important without adequately differentiating relationships between nodes. Second, some noisy nodes may interfere with accurate sentiment classification. This interference weakens the relationship between specific aspects and sentiment words, making it challenging to precisely articulate the connection between them. Third, some nodes in the dependency trees do not have direct dependency relationships but play critical roles in determining aspect-level sentiment polarities. For instance, as shown in Figure 1, the word “*powerful*” and aspect “*product*” are pivotal for deciding the sentiment polarity of the aspect, but there is no direct dependency between them, thus their important relationship can be easily overlooked. However, many existing studies have overlooked the relationships between nodes that are crucial for determining the sentiment relationship of specific aspects.

**Figure 2:**
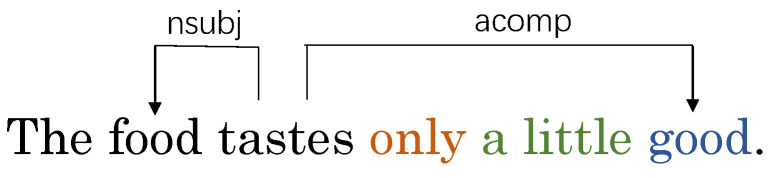
An example sentence of the ABSA task from the restaurant reviews, which illustrates the usefulness of the structured dependency tree in a sentence.

To address the first issue, in [8], attention scores were introduced as weight matrices, achieving favorable results. However, this method solely extracts information between nodes from a semantic perspective, neglecting to accurately represent syntactic relationships between nodes. Regarding the second issue, some methods reshape and prune dependency trees to alleviate the aforementioned problems, mainly by taking the aspect as the central node and retaining related nodes while removing irrelevant nodes [9,10]. This effectively extracts information between the aspect and key words. However, it also damages the syntactic dependency tree and leads to loss of syntactic information. Furthermore, key words without direct edges to the aspect may be pruned, resulting in missing information of pivotal key words. As shown in Figure 2, with the reshaped syntax structure, “*a little*” no longer modifies the adjective “*good*” and “*only*” no longer modifies “*a little*”, thus losing the negation meaning and causing incorrect positive sentiment prediction towards the aspect by solely depending on the opinion word “*good*”. On the other hand, the words “*only*”, “*a*”, and “*little*” do not have direct dependency edges to the aspect “*food*”, which may lead to pruning of pivotal words like “*only*”, “*a*”, “*little*” reresenting double negation and affect prediction of the aspect “*food*”’s sentiment. For the third issue, although some models take into account sub-dependency relationships between nodes [11], they cannot precisely capture pivotal word relationships and introduce more noise issues.

To address the aforementioned problems, this paper proposes a Structured Dependency Tree Graph Convolutional Network (SDTGCN) model to analyze sentiment orientations of specific aspects. Instead of reshaping and pruning the dependency tree, our model adjusts the edge weights between nodes based on positional information, sentiment commonsense knowledge, part-of-speech tags, and syntactic dependency distances. The SDTGCN model aims to explore relationships between crucial words. In a structured dependency tree, closer proximity to the aspect word generally has a greater impact, making position information influential in aspect-level sentiment analysis. Furthermore, sentiment common sense knowledge assigns different sentiment scores to words based on their emotional tone, helping identify the sentiment tendency of contextually important words. This assigns higher weights to emotionally stronger words and lower weights for weaker ones. By leveraging sentiment common sense knowledge, we can uncover the emotional tendencies of contextually important words. Moreover, we not only utilize part-of-speech tags to obtain pivotal word information, but also further leverage part-of-speech tags and dependency distances to discover relationships between nodes without direct dependencies yet playing significant roles in determining aspect sentiment polarities. If two words are both of crucial part-of-speech types for determining sentiment polarity and have a close syntactic dependency distance, higher weights can be assigned to strengthen the representation between these crucial words. For example, as shown in Figure 1, “*product*”, “*powerful*” and “*same*” are all crucial words for determining sentiment polarity. However, “*product*” and “*powerful*” have a close syntactic dependency distance, thus receiving higher weights to enhance their dependency relationship. Conversely, “*product*” and “*same*” have a distant syntactic dependency distance, resulting in lower weights and a weakened dependency relationship. Therefore, the structured dependency tree effectively preserves the syntactic relationships of the original text. Depending on the importance of each word, different weights are assigned to specific aspects and other words, allowing for a comprehensive expression of the relationship between specific aspects and contextual information, thereby enhancing the model’s performance. Finally, we adopt a weighted graph convolutional network to aggregate node information, aggregating different nodes based on their importance to the context during the process, which can obtain more precise final representations for aspects.

The main contributions of this paper are as follows:

1. We propose a structured dependency tree based on node weights, incorporating positional, sentiment commonsense, part-of-speech, and syntactic dependency distance information to enrich the generic dependency tree. This enables sufficient extraction of relationships between aspects and corresponding opinion words. We further aggregate node information using a weighted graph convolutional network.
2. We utilize part-of-speech tags and dependency distances to discover connections between pivotal nodes without direct dependency edges in the trees, thereby analyzing sentiment orientations of specific aspects.
3. Experimental results on five benchmark datasets demonstrate the effectiveness of our proposed method in aspect sentiment analysis, outperforming existing state-of-the-art approaches.

The remainder of this paper is organized as follows: Section 2 reviews related work. Section 3 elaborates the proposed model. Experimental setup and results are presented in Section 4. Finally, Section 5 concludes this paper and discusses future work.

## 2. Related Work

### 2.1. Aspect-Level Sentiment Analysis

Aspect-based sentiment analysis has become a hot research topic, as it can effectively capture relationships between aspect and opinion words and extract fine-grained sentiment orientations. In recent years, numerous methods have been proposed for modeling aspect sentiment analysis. Among them, many researchers leverage neural networks to represent aspect information in sentences. Dong et al. employ RNNs to represent semantic information in sentences [3]. Tang et al. utilize two LSTMs to capture relationships between the aspect and its left/right contexts separately [4]. Majumder et al. identify aspects first using a BiRNN-CRF model, and then enrich word embeddings with bidirectional GRU hidden states for detecting aspect sentiment polarities [12]. The above models build sequential information of sentences, while attention-based models can focus on pivotal parts of sentences. Some researchers employ an attention-based LSTM model concentrating on relevant key information to the aspect [5,13]. Ma et al. introduce two attention mechanisms for information interaction between the aspect and contexts [14]. Gu et al. incorporate position information to assign higher weights to contexts closer to the aspect, and then apply a bidirectional attention mechanism to extract relationships between the specific aspect and contexts [15]. Some methods also utilize attention mechanisms, semantic relevance, etc., to extract semantic information between the aspects and contexts in sentences [16,17]. Additionally, convolutional neural networks have been widely used in sentiment analysis tasks. Xue et al. extract sentiment features from text using CNNs and gating mechanisms and overcome LSTM sequence dependency and structural complexity by max pooling along the sequence direction to filter unimportant sentiment information [18]. Ayetiran presents an attention-based deep learning technique using CNNs and BiLSTMs to extract high-level semantic features, which can effectively capture contextual feature representations of text [19]. These models demonstrate that deep neural networks can obtain effective deep representations of sentences for aspect-level sentiment classification.

### 2.2. Graph Neural Networks

With the development of deep learning, graph neural networks have become a hot research area, as they can represent non-structural data. Some surveys provide a detailed introduction to the application of aspect-level sentiment analysis in graph convolutional networks [20,21,22]. Some researchers construct a dependency tree using a dependency parser and feed it into a graph convolutional network, which can sufficiently acquire the sentence’s syntactic knowledge for extracting aspect-specific sentiment orientations. The graph structure effectively extracts syntactic information from high-order neighborhoods, further optimizing the accuracy and generalization performance of sentiment classification compared to traditional neural network models [6,23,24]. Wang et al. propose a new method to encode the syntactic information of sentences, primarily taking the aspect as the root node, performing pruning and modification on the dependency tree, and then utilizing dependency labels to construct a relational graph attention model for further expressing relationships between the aspect and contexts. The method shortens the distance between specific aspect words and opinion words, enhances the representation of specific aspects, and utilizes dependency labels in the graph attention network to focus more on nodes and their relationships that significantly impact the graph, thereby further optimizing the representational capacity of the graph structure [9]. Liang et al. integrate sentiment knowledge into the syntax dependency tree, which is then fed into a graph convolutional network to extract neighbor information. Compared to traditional syntactic dependency graphs, this method more comprehensively considers complex relationships between nodes in the text using commonsense knowledge, effectively strengthening the relationship between specific aspects and opinion words and achieving better performance improvement [17]. Veyseh et al. introduce a novel aspect-level sentiment classification model based on gated graph convolutional networks. The key idea of the model is to use a gating mechanism to filter out irrelevant information, thereby uncovering crucial information related to aspect words. This method not only improves the accuracy of information extraction, but also helps the model gain a more comprehensive understanding of important information related to specific aspects in the text, thereby enhancing its performance in relevant tasks [25]. Zhao et al. construct a graph taking aspects as nodes, and apply graph convolutional networks to extract inter-aspect sentiment dependencies for analyzing different aspects. By exploring the interaction of information between different aspect words, this method provides the model with more expressive features [26]. Lu et al. add a new gate on the basis of the LSTM to control the influx of irrelevant contextual information to the aspect, and then input it into graph convolutional networks for more precise aspect representations. This method effectively improves sentiment classification performance by extracting key information [27]. Xu et al. build a heterogeneous graph with sentence information, aspect nodes and context, and utilize graph convolutional networks to acquire their relationships, thereby sufficiently representing specific aspects. This method enriches traditional single-syntax representations by constructing a heterogeneous graph with multi-faceted information and achieves significant improvement [28]. Zhou et al. leverage syntactic and knowledge graphs to express relationships between aspects and contexts, and feed them into graph convolutional networks to update neighbor node information. This method incorporates common sense knowledge to enhance the semantic knowledge of sentences, complementing ordinary syntax structures and achieving further improvement [29]. Li et al. construct a syntactic graph convolutional model based on dependency parsers and a semantic graph convolutional model based on self-attention, then effectively fuse them with BiAffine to extract pivotal information from sentences. This method precisely models specific aspect words from both syntactic and semantic perspectives, achieving satisfactory performance improvement [8]. Ke et al. present a novel Syntactically Dependent ATTention model (SDATT) that aims to explore context-aspect relationships by incorporating syntactic distances and dependency paths through dependency trees, paying more attention to aspect-relevant contexts and generating more robust aspect representations [30]. Zhang et al. integrate syntactic, semantic knowledge, and context graphs into one SSC graph, feed it into graph convolutional networks to obtain representations for specific aspects, and perform aspect sentiment analysis using an attentive CNN with positional embeddings. This method extracts key information from sentences from different perspectives, such as syntax, semantics, and knowledge, effectively fusing them through a convolutional neural network, yielding good results [31]. Lu et al. propose a novel graph convolutional network with sentiment interactions and multi-graph perception, simultaneously considering complementarity between semantic dependencies and sentiment interactions [32]. In addition, they also build a multi-graph perception mechanism to capture specific inter-graph dependency relationships, thus reducing overlapping information and effectively improving classification performance. Zhang et al. propose a novel syntax and semantics-enhanced graph convolutional network (SSEGCN) model for ABSA tasks. The architecture integrates semantic and syntactic information, combining attention score matrices with syntax mask matrices to fuse semantic and syntactic information, achieving better performance [33]. Liang et al. design a structure-enhanced interactive model (SE-IAN-G) for aspect-level sentiment classification. The main idea is that structurally enhanced representation eliminates the negative impact of certain words, making the expression of target words and context words more closely connected, a relationship ignored by other methods [34].

Overall, we summarize and compare the aforementioned models in Table 1. From the table, it can be observed that most current mainstream approaches effectively enhance aspect-level sentiment analysis performance by integrating syntactic knowledge, common-sense knowledge, and graph neural networks in deep learning. However, the majority of existing models only utilize simple syntactic dependency trees to extract sentence syntactic knowledge or reshape dependency trees. Few models enrich the syntactic dependency tree from multiple perspectives to extract more accurate node representations. Furthermore, although part-of-speech information has been studied by many researchers, there are few methods that combine part-of-speech with syntactic dependency distance to capture relationships between important nodes that lack direct dependency connections.

## 3. The Proposed Method

Utilization of different types of information in sentences, such as aspect positions, sentiment relationships, part-of-speech tags, and dependency distances, can lead to more accurate results in sentiment analysis. As discussed previously, on the one hand, we need to find nodes more relevant to the aspect while retaining syntactic information, avoiding the loss of information caused by pruning. On the other hand, we also need to discover sentiment relationships between nodes without direct dependencies in order to uncover potential sentiment information. Therefore, this paper constructs two structured dependency weight matrices using various sentence information—the adjacency-enhanced dependency weight matrix and the subadjacent dependency weight matrix. This is to fully extract hidden sentiment information in sentences from different perspectives. Specifically, the adjacency-enhanced dependency weight matrix assigns different weights to dependencies between words based on positional and sentiment common sense knowledge, on top of the generic dependency tree constructed by dependency parsers. This describes the relevance between the nodes better. The subadjacent dependency weight matrix utilizes pivotal part-of-speech tags and dependency distances to construct subadjacent dependency edges, uncovering sentiment propagation relationships between nodes without direct dependencies.

**Figure 3:**
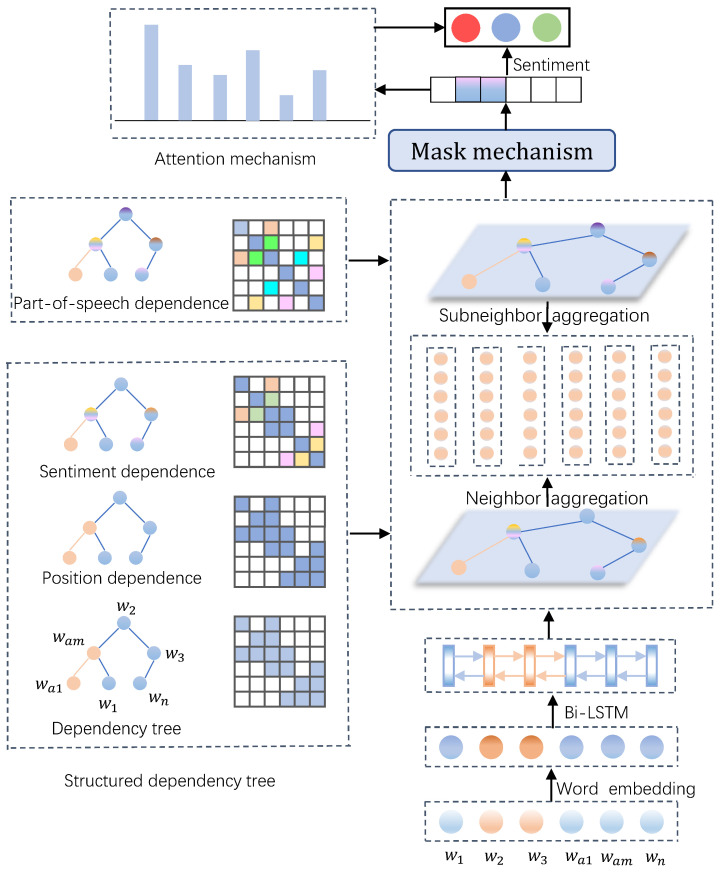
Overall architecture of the proposed SDTGCN model.

After obtaining the structured dependency tree, we utilize a weighted graph convolutional network to aggregate node information, effectively distinguishing the importance of nodes, thereby aggregating information from important nodes with larger weights and information from secondary nodes with smaller weights. Finally, we fuse them through masking and attention mechanisms to acquire the final sentiment orientation of the specific aspect. Some notations are provided to clearly explain the proposed model, as shown in Table 2. The overall framework of our model is illustrated in Figure 3.

### 3.1. Definitions

Aspect-level sentiment analysis basically depends on context information to predict the sentiment polarity of particular aspects. Given a sentence contains *n* words $s=\{w_{1},w_{2},\ldots ,w_{a1},w_{a2},\ldots ,w_{ak},\ldots ,w_{n}\}$, where aspect sequence $s_{a}=\left\{w_{a1},w_{a2},\ldots ,w_{ak}\right\}$ is the subsquence of *s*, $w_{i}$ denotes the *i*th context word, and $w_{ai}$ denotes the *i*th aspect.

### 3.2. Text Representation

#### 3.2.1. Word Embedding

For the input text, we obtain high-dimensional embedded representations of the sentence using a pretrained GloVe embedding matrix, where each word in the sentence is mapped to vector $x_{i}\in R^{d_{w}}$, where $d_{w}$ denotes the dimension of word vectors. After embedding, we can obtain embedding matrix $X=\{x_{1},x_{2},\ldots ,x_{a1},x_{a2},\ldots ,x_{\mathrm{ak}},\ldots ,x_{n}\}$ for the input sentence, where $X\in R^{d_{w}\times n}$, *n* is the length of the sentence, $x_{i}$ denotes the embedding of the *i*th context word, $x_{\mathrm{ai}}$ denotes the embedding of the *i*th aspect.

#### 3.2.2. Bi-LSTM Embedding

To obtain contextual information of the sentence, we feed the word embedding matrix into a Bi-LSTM, using a forward LSTM and a backward LSTM to capture bidirectional contextual information. After Bi-LSTM embeddings, we obtain forward hidden state ${\vec{h}}_{i}\in R^{d_{h}}$ and backward hidden state ${\overset{\leftarrow }{h}}_{i}\in R^{d_{h}}$, where $d_{h}$ is the number of hidden units. Finally, we concatenate the forward and backward states to obtain the final hidden representation H for each position.

### 3.3. Structured Dependency Trees

In this paper, we construct structured dependency trees on top of the generic dependency tree by incorporating position information, sentiment common sense knowledge, part-of-speech tags, and syntactic dependency distances. This allows sufficient representation of the aspects and contexts while retaining syntactic knowledge, enabling thorough extraction of potential sentiment information. The structured dependency tree mainly consists of an adjacency-enhanced dependency tree and a subadjacent dependency tree, which are used to uncover hidden information from two perspectives—the dependency relationships of individual aspects, and the sentiment relationships between aspects and pivotal words.

Specifically, the adjacency-enhanced dependency tree assigns weights to edges between words in the generic dependency tree based on position and sentiment commonsense knowledge to differentiate the importance of context words to the aspect. The subadjacent dependency tree builds connections between pivotal words and the aspect based on part-of-speech tags and dependency distances, uncovering hidden sentiment propagation paths. By combining the two sub-structures, the structured dependency tree can sufficiently preserve syntactic relationships while emphasizing important sentiment signals.

To better leverage graph convolutional networks, we first construct a dependency tree for each sentence using a dependency parser to obtain syntactic knowledge of the sentence. Adjacency matrix D of the dependency tree is defined as follows:

(1) $$D_{\mathrm{ij}}=\left\{\begin{array}{cc} 1, & i=j\;\mathrm{or}\;w_{i},w_{j}\;\mathrm{contains}\;\mathrm{dependency},\\ 0, & \mathrm{otherwise}, \end{array}\right.$$

where $D_{\mathrm{ij}}$ is the dependency matrix, $w_{i}$ denotes the *i*th context word.

#### 3.3.1. Adjacency Enhanced Dependency Weight Matrix

Context words at different positions relative to the aspect have varying degrees of influence on aspect sentiment analysis. Context closer to the aspect has a larger impact, with influence decreasing as distance increases. Thus, we construct a position weight matrix to enhance the generic adjacency matrix by incorporating the positional information of each node’s distance to the aspect node. Position weight matrix P is implemented as follows:

(2) $$P_{\mathrm{ij}}=\left\{\begin{array}{cc} 1, & w_{i}\in s_{a}\;\mathrm{and}\;w_{j}\in s_{a},\\ \frac{1}{|j-p_{a}|+1}, & w_{i}\in s_{a},\\ \frac{1}{|i-p_{a}|+1}, & w_{j}\in s_{a},\\ \frac{1}{2(|i-p_{a}|+1)}+\frac{1}{2(|j-p_{a}|+1)}, & \mathrm{otherwise}, \end{array}\right.$$

where $p_{a}$ is the start position of the aspect in the sentence, $w_{i}$ denotes the *i*th word in the sentence, and $s_{a}$ is the set of aspects in the sentence.

To utilize sentiment common sense knowledge between the aspects and context words, we obtain the sentiment scores of each word from the common sense knowledge library SenticNet [35]. Different weights are then assigned to each node accordingly. SenticNet is a knowledge base of common sense information that includes 100,000 concepts related to rich emotional attributes (e.g., emotion, polarity, semantics). These emotional attributes provide conceptual-level representations and semantic connections for various aspects and their associated sentiments. The sentiment common sense knowledge library assigns sentiment scores to opinion words, which can effectively indicate the sentiment orientation of words. Specifically, positive words have scores close to 1, negative words have scores close to −1, and neutral words have scores close to 0. We construct a sentiment knowledge matrix E based on this knowledge, which is defined as

(3) $$E_{\mathrm{ij}}=\left\{\begin{array}{cc} S(w_{i},w_{j}), & w_{i}\in s\;\mathrm{and}\;w_{j}\in s,\\ 0, & \mathrm{otherwise}, \end{array}\right.$$

(4) $$S\left(w_{i},w_{j}\right)=S_{w_{i}}+S_{w_{j}},$$

where $S_{w_{i}}$ denotes the sentiment score of $w_{i}$, $S(w_{i},w_{j})$ is the sentiment weight between $w_{i}$ and $w_{j}$.

In order to emphasize the importance of specific aspects, we assign them higher weights. The formula for the aspect matrix A is as follows:

(5) $$A_{\mathrm{ij}}=\left\{\begin{array}{cc} 1, & w_{i}\in s_{a}\;\mathrm{or}\;w_{j}\in s_{a},\\ 0, & \mathrm{otherwise}. \end{array}\right.$$

Finally, we can obtain the representation of the adjacency enhanced dependency weight matrix $W_{\mathrm{adj}}$ as follows:

(6) $$W_{\mathrm{adj}}=D⊙\left(P+E+A\right).$$

#### 3.3.2. Subadjacent Dependency Weight Matrix

Aspect-based sentiment analysis focuses on sufficiently expressing relationships between aspects and opinion words. We find that part of speech can effectively uncover sentiment orientations between specific aspects and opinion words. As shown in Figure 1, the sentiment polarity of the aspect “*product*” is determined to be negative directly by the negation adverb “*not*”. Through dataset comparison, we observe that most opinion words consist of adjectives, adverbs, noun phrases, etc., while conjunctions are often overlooked. However, some conjunctions can play pivotal roles. In Figure 1, the adverb “*not*” determines the aspect “*product*” as negative, while although the opinion word “*same*” modifying the aspect “*performance*” does not have explicit sentiment, the coordinating conjunction “*and*” indicates “*performance*” shares the same negative polarity with “*product*” based on their paratactic relationship. This demonstrates that conjunctions can also significantly impact judgment of aspect sentiment orientations. Therefore, we also consider conjunctions as pivotal words to uncover key sentiment signals.

Most existing methods simply embed all word part-of-speech (POS) tags to enrich word representations while overlooking pivotal POS tags and relationships between them [10]. However, words with shorter dependency distances to the aspect have larger influence on it. To uncover connections between pivotal words without direct dependencies yet playing vital roles in determining aspect sentiment, we combine POS tags and dependency distances to discover relationships between such pivotal words. Different weights are then assigned as subadjacent edges to construct the subadjacency matrix. In this work, we not only consider pivotal POS tags, but also incorporate dependency distances to selectively highlight relationships between pivotal words, even without direct dependencies in the original tree. Words closer to the aspect are more likely to modify its sentiment.

We define pivotal={adjectives,adverbs,nounphrases,conjunctions,aspects}. If two words both belong to the pivotal set, the closer their syntactic dependency distance, the greater the edge weight between them. Subadjacency matrix $W_{\mathrm{sub}}$ can be expressed as follows:

(7) $$W_{\mathrm{sub}}\left(i,j\right)=\left\{\begin{array}{cc} 2-\frac{d_{ij}}{n}, & w_{i}\in pivotal\;\mathrm{and}\;w_{j}\in pivotal,\\ 1-\frac{d_{ij}}{n}, & w_{i}\in pivotal\;\mathrm{or}\;w_{j}\in pivotal,\\ 0, & \mathrm{otherwise}, \end{array}\right.$$

where $d_{ij}$ denotes the dependency distance between *i* and *j*, and *n* is the number of the words in the sentence.

### 3.4. Weighted Aggregation Graph Convulutional Network

**Figure 4:**
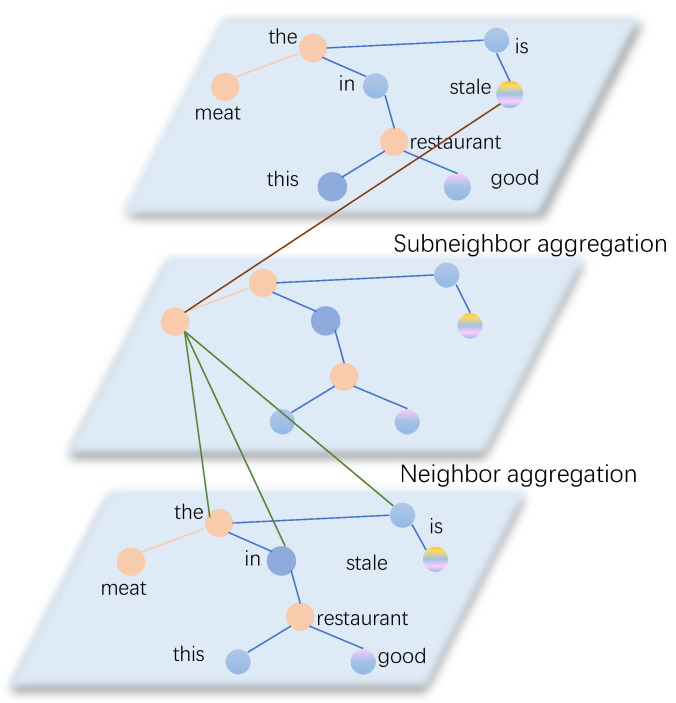
The weighted aggregation graph convolution of target node “*stale*”. The green line indicates the neighbor aggregation edge, and the brown line indicates the subconnection aggregation edge.

In this study, we employ a weighted aggregation graph convolutional network that takes into account the varying importance of different nodes. Instead of treating all nodes equally, our approach assigns higher attention to pivotal nodes and lower attention to less critical ones. Figure 4 illustrates the aggregation process for the node “*meat*”. Initially, the node “*meat*” aggregates its adjacency enhanced nodes such as “*the*”, “*is*”, and “*restaurant*” to gather syntactic information from the dependency tree. Subsequently, it aggregates “*stale*”, which represents subadjacent nodes, to capture the relationships among non-directly dependent key nodes.

Within the SDTGCN model, adjacency-enhanced weight matrix $W_{\mathrm{adj}}$ and subadjacency weight matrix $W_{\mathrm{sub}}$ from the structured dependency tree, along with the hidden state information H obtained from BiLSTM, serve as inputs. These inputs are fed into the weighted aggregation graph convolutional network to obtain adjacency-enhanced information and subadjacency information for each word. In each layer of the aggregation graph convolutional layer, the *i*th node’s hidden information $h^{s}\left(i\right)$ is updated based on its adjacency-enhanced nodes and subadjacent nodes:

(8) $$h_{l}^{n}\left(i\right)=\mathrm{ReLU}\left(\left[H_{l-1}^{n}{\tilde{W}}_{\mathrm{adj}}⊕h_{l-1}^{n}\left(i\right)\right]M_{\mathrm{adj}}+b_{l}\right),$$

(9) $$h_{l}^{s}\left(i\right)=\mathrm{ReLU}\left(\left[H_{l}^{n}{\tilde{W}}_{\mathrm{sub}}⊕h_{l-1}^{s}\left(i\right)\right]M_{\mathrm{sub}}+b_{l}\right),$$

where $H_{l-1}$ is the representation matrix of the l−1 layer, $H_{l}^{n}$ is the neighbor feature matrix of *l*-th layer, $M_{\mathrm{adj}}$ and $M_{\mathrm{sub}}$ are the parameter matrices; they are updated during the training process, ${\tilde{W}}_{\mathrm{adj}}$ and ${\tilde{W}}_{\mathrm{sub}}$ are the normalized matrices of $W_{\mathrm{adj}}$ and $W_{\mathrm{sub}}$, respectively, $b_{l}$ is the bias, and ⊕ denotes the concatenation operation.

We construct a concrete aspect masking mechanism to retain the aspect representations in the final GCN output while masking out non-aspect word representations in the sentence. This mechanism can effectively acquire valid information about specific aspects to determine their sentiment orientation. The final representation of the sentence is

(10) $$H^{c}=\left\{0,0,0,\ldots ,h^{s}\left(a_{1}\right),h^{s}\left(a_{2}\right),\ldots ,h^{s}\left(a_{k}\right),0,0,\ldots ,0\right\}.$$

### 3.5. Atterntion Mechanism

We leverage an attention mechanism to extract deep features that capture the relationship between specific aspects and contextual information. We employ an index-based attention mechanism for the hidden state information obtained from BiLSTM and the representations generated using the aspect masking mechanism:

(11) $$\beta_{t}=\sum_{j=1}^{n}h^{c}{\left(i\right)}^{T}h^{c}\left(j\right),$$

(12) $$\alpha_{t}=\frac{\exp\left(\beta_{t}\right)}{\sum_{1}^{n}\exp\left(\beta_{i}\right)},$$

(13) $$\gamma =\sum_{t=1}^{n}\alpha_{i}h^{c}\left(i\right),$$

where $\beta_{t}$ is utilized to model the relationship between aspects and their context, $\alpha_{t}$ represents attention weights, and γ signifies index-based attention representation. Finally, we feed the ultimate representation of the sentence into a classifier for sentiment analysis.

(14) $$y=\mathrm{softmax}(W_{p}\gamma +b_{p}),$$

where softmax() function is applied to learn the output distribution of the sentiment classifier, y denotes the predicted sentiment distribution, $W_{p}$ and $b_{p}$ are the trained parameters.

### 3.6. Training

To minimize the loss function, we employ minimum cross-entropy regularization to train the model and utilize the standard gradient descent algorithm to optimize and update the proposed model’s parameters:

(15) $$L=-\sum^{N}y_{i}\log\left({\hat{y}}_{i}\right){\left||\theta |\right|}^{2},$$

where *N* represents the number of samples in the dataset, $y_{i}$ denotes the ground truth values, ${\hat{y}}_{i}$ represents the predicted values, θ encompasses all training parameters, and λ is the coefficient for $L_{2}$ regularization.

## 4. Expriments

### 4.1. Datasets and Setting

To validate the effectiveness of our proposed model, we conduct experiments on five public datasets: Lap14, Rest14, Rest15, Rest16, and Twitter. These datasets are sourced from various SemEval tasks, including SemEval 2014 task 4 (Lap14, Rest14) [36], SemEval 2015 task 12 (Rest15) [37], and SemEval 2016 task 5 (Rest16) [38]. The SemEval datasets are divided into two main categories, namely Restaurant and Laptop. Each dataset consists of both training and testing sets, with each review sentence containing one or more aspects and their corresponding sentiment polarities, which are categorized as positive, neutral, and negative. Dataset representations are provided in Table 3 for reference.

In our experiments, we initialize the word embeddings with 300-dimensional pre-trained GloVe vectors. The hidden state vectors for all neural network layers are set to 300 dimensions. All weight matrices are initialized from a uniform distribution U(−0.01,0.01). The Adam optimizer with a learning rate of 0.001 is utilized to optimize and update all models. The $L_{2}$ regularization coefficient λ (learning rate) is set to 0.00001, and the batch size is set to 32. The number of layers in SDTGCN is set to 1. We obtain results over 20 random initializations and evaluate the model performance using accuracy and Macro-F1 as evaluation metrics.

### 4.2. Baseline Models

To evaluate the efficacy of our model, we make comparisons with the following baseline models on five datasets:

- **TD-LSTM** [39]: The TD-LSTM model employs two target-dependent LSTM networks to capture dependencies between specific aspects and left and right contexts separately.
- **ATAE-LSTM** [5]: The ATAE-LSTM model utilizes an attention-based LSTM model to compute attention scores for specific aspects, thus enabling the model to focus on pivotal contextual information around different aspects in the sentence.
- **IAN** [14]: The IAN model uses two interactive attention networks to learn representations of contexts and targets, which allows for focusing on pertinent parts of contexts and targets by utilizing inter-attention. It generates aspect-specific representations for contexts and targets separately.
- **MGAN** [40]: The MGAN model proposes a fine-grained attention mechanism that can capture word-level interactions between aspects and contexts.
- **MemNet** [4]: This model develops a deep memory network to capture pertinent contextual information for aspect-level sentiment classification. Compared to RNN models like LSTM, this approach is simpler and faster.
- **AOA** [41]: This model captures interactions between context and aspects via an attention mechanism that focuses on salient parts of the sentence.
- **TNet-LF** [42]: This model utilizes CNN layers to extract pertinent features based on LSTM layers from transformed lexical representations.
- **ASCNN** [7]: This model simplifies ASGCN by substituting two CNN layers for the two GCN layers in ASGCN.
- **R-GAT** [9]: The R-GAT model defines an aspect-oriented dependency tree structure rooted at the target aspect by reshaping and pruning the original dependency tree. It then leverages graph attention networks to encode the new tree and analyze the sentiment orientation of specific aspects.
- **SK-GCN** [29]: This model employs a novel syntax and knowledge-based graph convolutional network for aspect-level sentiment classification, primarily by modeling syntactic dependency trees and common sense knowledge graphs to enhance sentence representations for given aspects.
- **CDT** [6]: The CDT model simply aggregates GCN and BiLSTM models, demonstrating convolutional operations of GCNs on dependency trees to distill BiLSTM embeddings, thereby effectively capturing both structural and contextual information of sentences.
- **ASGCN** [7]: The ASGCN model constructs a dependency graph for each sentence and extracts syntactic information and word dependencies via graph convolutional networks.
- **BiGCN** [43]: This model proposes a novel hierarchical architecture of lexical and syntactical graphs. It utilizes a global word-level graph to encode co-occurrence information of words, and separate hierarchical syntax to distinguish various types of dependency relationships or word pair relations.
- **AGCN** [11]: This model introduces two aggregating functions to iteratively update each node’s representation from its neighborhood and leverage sub-dependencies of nodes to incorporate more relevant node information.
- **RMN** [44]: The RMN model proposes an innovative relation-constructing multitask learning network that generates aspect representations via graph convolutional networks with semantic dependency graphs and acquires relationships between aspects for sentiment classification.
- **InterGCN** [45]: This work introduces a novel interactively graph-perceiving model based on graph convolutional networks for sentiment analysis by constructing a heterogeneous graph for each example using aspect-oriented and inter-aspect contextual dependencies.
- **GL-GCN** [46]: This model concurrently introduces global and local structural information in aspect-based tasks to sufficiently extract accurate representations of specific aspects and contexts.
- **SenticGCN** [17]: The SenticGCN model aggregates sentiment knowledge from SenticNet to construct graph neural networks, enhancing dependency graphs of sentences. The novel sentiment-enhanced graph model can accurately acquire distinct affective features of different aspects and fully capture relationships between specific aspects and contextual information.

### 4.3. Results and Analysis

As shown in Table 4, we present the performance of all baseline models and our proposed model on the five public datasets, i.e., Lap14, Rest14, Rest15, Rest16, and Twitter. The table allows us to draw the following conclusions:

First, compared to the baseline models, including sequence-based models, self-attention models, convolutional neural network models, and graph neural network models, the SDTGCN model outperforms the baseline models on the Rest14, Rest15, Rest16, and Twitter datasets. This effectively demonstrates the effectiveness of our proposed model in aspect-based sentiment analysis.

Second, the table reveals that our model outperforms syntatic models based on general dependency trees like CDT and ASGCN. This indicates that the structured dependency tree proposed in this paper enriches node information, allowing for it to better capture the sentiment dependencies between aspects and context information. This enrichment effectively avoids irrelevant context information as clues for determining the sentiment polarity of specific aspects.

Third, compared to conventional graph neural network models such as ASGCN, SDGCN, and SK-GCN, our model exhibits superior performance. This suggests that our proposed weighted aggregation graph convolutional network is capable of aggregating information from neighbor nodes and subadjacent nodes based on their respective importance levels. It assigns higher weight to pivotal nodes and lower weight to irrelevant noise nodes, thereby enhancing model performance.

### 4.4. Ablation Study

**Figure 5:**
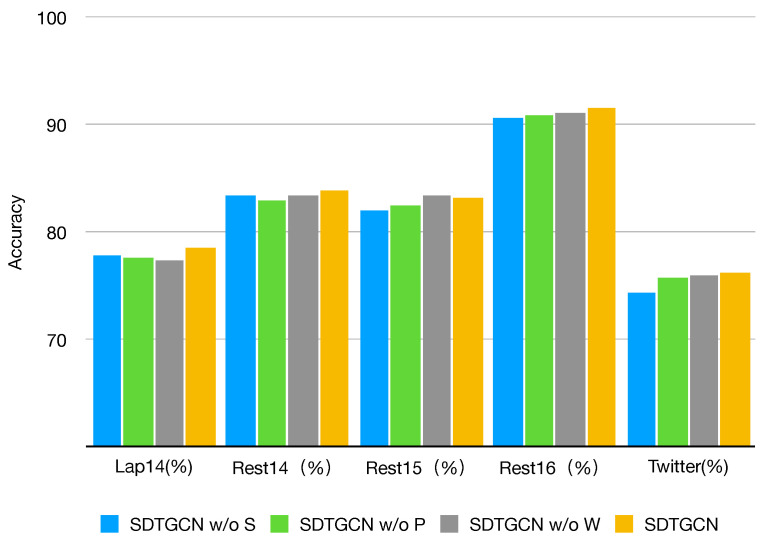
Ablation study on accuracy.

**Figure 6:**
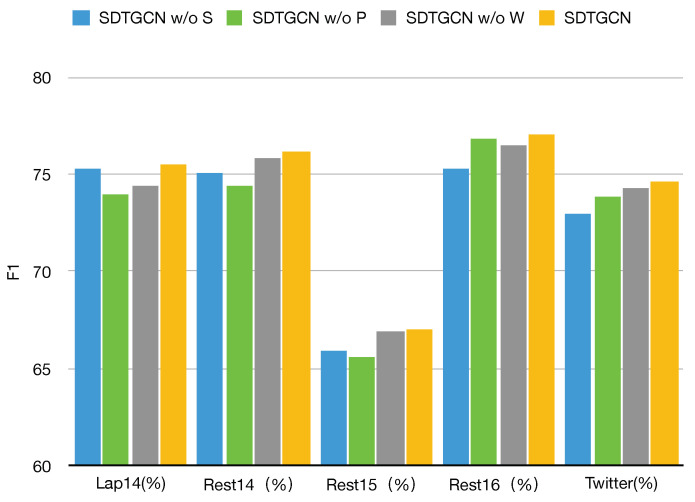
Ablation study on F1.

To evaluate the performance of different components in our proposed SDTGCN model, we conducted ablation experiments on the same five public datasets, as demonstrated in Figure 5 and Figure 6.

- SDTGCN w/o S: This represents the model with the structured dependency tree module removed, making it unable to fully investigate the relationships between aspects and context in sentences. As shown, there is a significant decrease in performance across all datasets when the structured syntax dependency tree module is removed compared to the SDTGCN model. This indicates that the structured syntax dependency tree enriches the general dependency tree, strengthens the contextual information in sentences, and effectively extracts and identifies specific aspect information.
- SDTGCN w/o P: This indicates the model with the subadjacent module that considers part-of-speech and syntactic dependency distance removed. As observed, removing this subadjacent module results in a relatively minor decrease in performance compared to the SDTGCN model. This suggests that considering part-of-speech and syntactic distance can effectively explore relationships between important nodes that lack direct dependencies, leading to noticeable improvements in aspect-based sentiment analysis.
- SDTGCN w/o W: This represents the model with the weighted aggregation graph convolutional network removed, which means it does not effectively aggregate information from adjacency-enhanced matrices and subadjacent matrices. Instead, it uses a regular graph convolutional network to aggregate neighbor node information. It is evident that removing the weighted aggregation graph convolutional network results in a relatively modest decline in performance compared to the SDTGCN model. This indicates that the weighted aggregation graph convolutional network plays a certain role in the SDTGCN model by aggregating node information based on importance levels, thereby enhancing the accuracy of node representations.

These ablation experiments demonstrate the significant contributions of the individual components within the SDTGCN model to its overall performance, reaffirming the model’s effectiveness in aspect-based sentiment analysis.

### 4.5. Study on Model Depth

**Figure 7:**
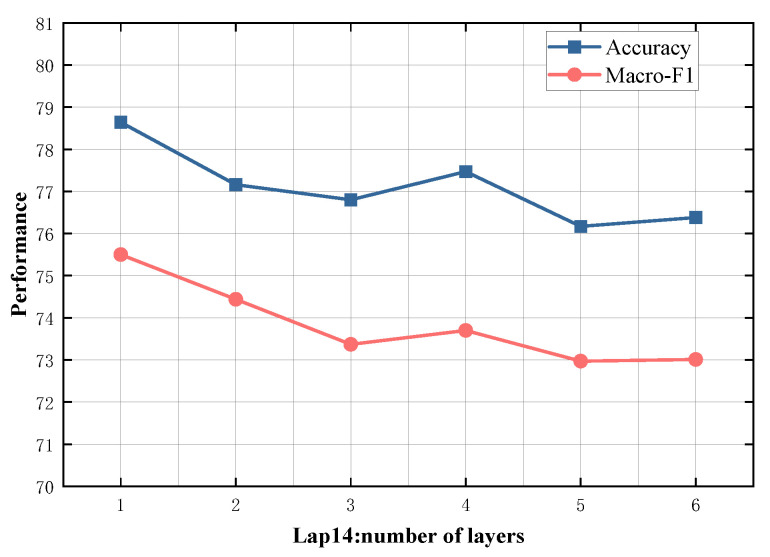
Model depth study on Lap14.

**Figure 8:**
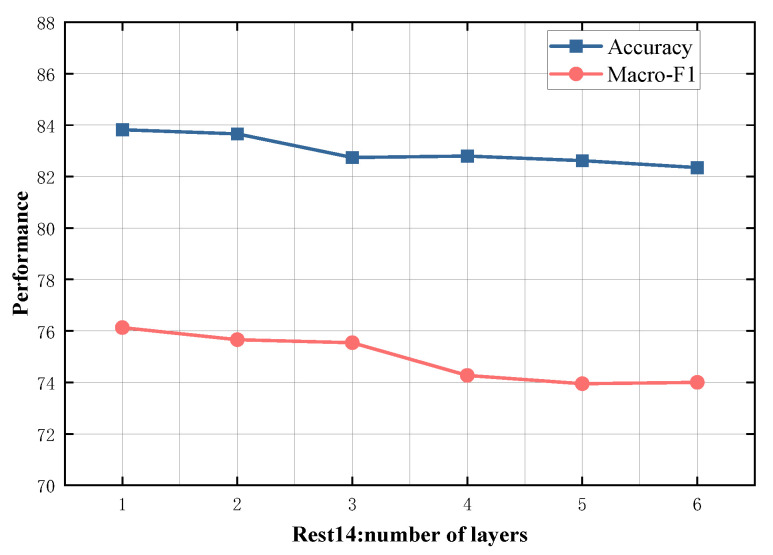
Model depth study on Rest14.

**Figure 9:**
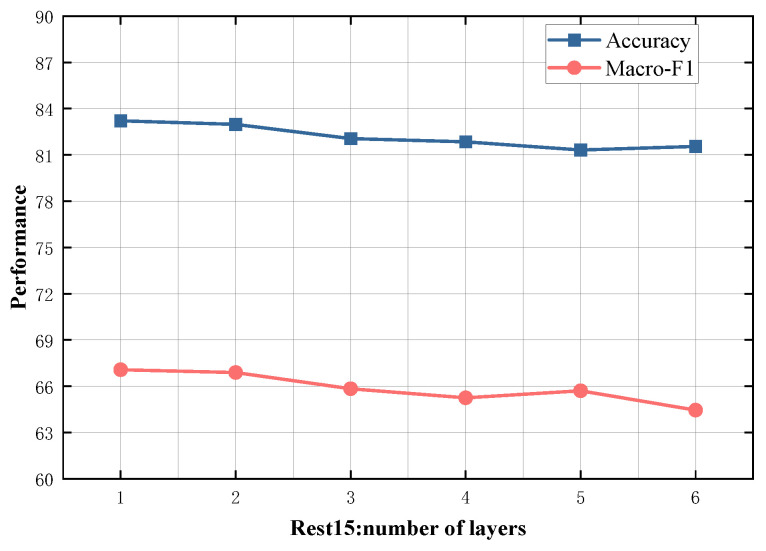
Model depth study on Rest15.

**Figure 10:**
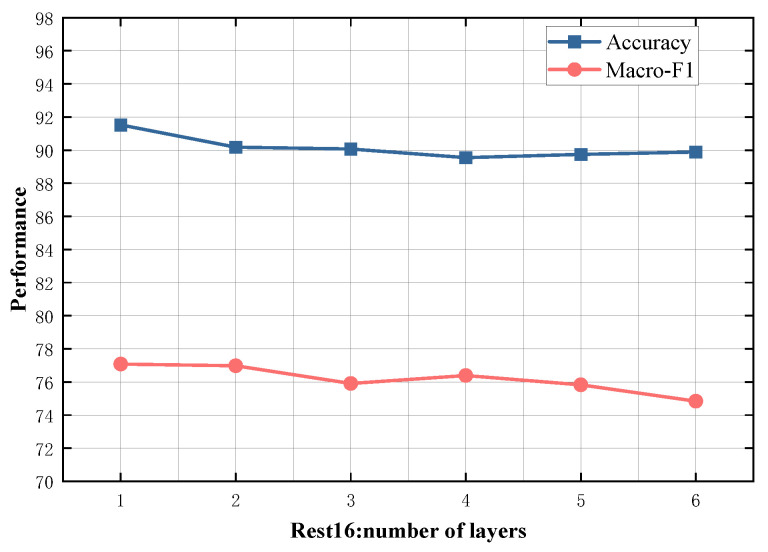
Model depth study on Rest16.

**Figure 11:**
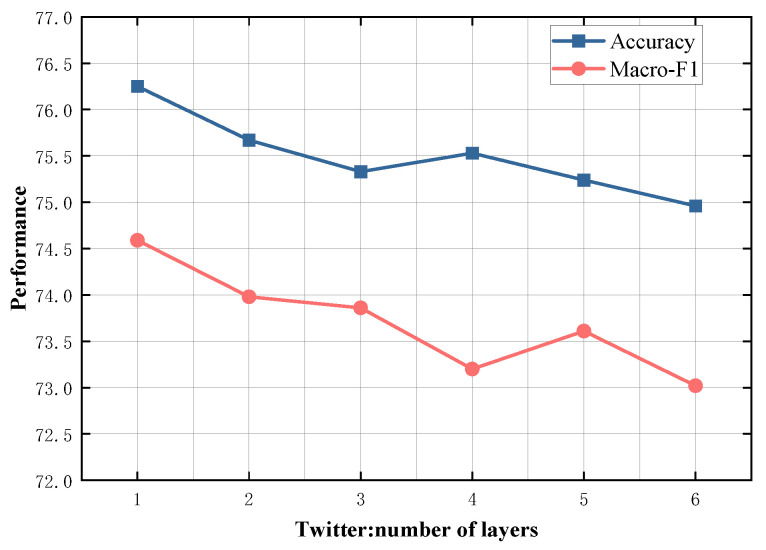
Model depth study on Twitter.

To evaluate the impact of different numbers of SDTGCN layers, we experiment with SDTGCN layers ranging from 1 to 6, and the results are presented in Figure 7, Figure 8, Figure 9, Figure 10 and Figure 11. From the graph, we can observe that a single layer of SDTGCN achieves the best performance, outperforming other numbers of SDTGCN layers. Therefore, we set the number of SDTGCN layers to 1 for our experiments. When the number of SDTGCN layers exceeds 1, the model’s performance in terms of accuracy and macro metrics shows diminishing returns. This may be because a higher number of layers introduces complexity that hinders the effective propagation of adjacency information. When the number of layers in SDTGCN is set to 1, the aggregated graph convolutional network first performs the initial aggregation on the enhanced adjacency matrix. Subsequently, it conducts a second aggregation on the sub-adjacency matrix based on the results of the first aggregation. Therefore, when the graph convolutional network performs one convolutional operation, it is equivalent to aggregating information from the nodes twice. This enables the extraction of sufficient neighboring node information while avoiding the introduction of unnecessary redundancy.

### 4.6. Case Study

As shown in Table 5, for the first example with the aspect “*screen*”, the attention-based model incorrectly focuses on the opinion word “*good*” and ignores the negation “*not*”, leading to an incorrect positive sentiment for the aspect. In contrast, both ASGCN and SDTGCN models accurately capture the dependency relationships of the aspect “*screen*” with both “*good*” and “*not*”, enabling them to correctly analyze the aspect’s sentiment as positive. This example demonstrates that dependency tree-based GCN models can flexibly capture sentence syntax and sentiment dependencies.

In the second example, the ASGCN model incorrectly identifies the sentiment polarity of the aspect “*food*”, whereas the SDTGCN model accurately recognizes the sentiment polarity of “*food*”. This discrepancy is attributed to the SDTGCN model’s structured dependency tree, which enriches the general syntax dependency graph. It does so by incorporating location information, sentiment knowledge, part-of-speech, syntactic dependency distance, among others, to enhance the importance of aspects with opinion words while weakening connections between irrelevant words. This results in a more precise expression of the sentiment dependencies between specific aspects and opinion words.

In the third example, both Attention and ASGCN models fail to correctly identify the sentiment polarity of the aspect “*meat*”. In contrast, our proposed SDTGCN model accurately identifies the sentiment polarity of “*meat*”. The reason for the misclassification by Attention and ASGCN models is that they do not consider the relationship between key words “*meat*” and “*stale*”, which lack direct dependency. Therefore, they fail to correctly identify the aspect’s sentiment polarity. In this case, compared to the opinion word “*stale*”, the aspect “*meat*” has a closer syntactic dependency distance to the opinion word “*good*”, indicating a negative sentiment. The SDTGCN model accounts for the influence of part-of-speech and syntactic dependency distance, effectively mining the relationships between pivotal nodes that lack direct dependencies, thereby enhancing aspect-based sentiment analysis performance.

### 4.7. Visualization of the SDTGCN

**Figure 12:**
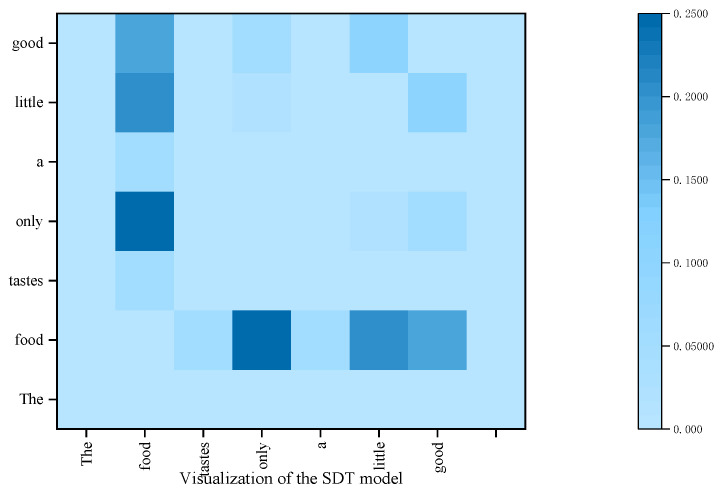
Attention weights of the SDTGCN model.

**Figure 13:**
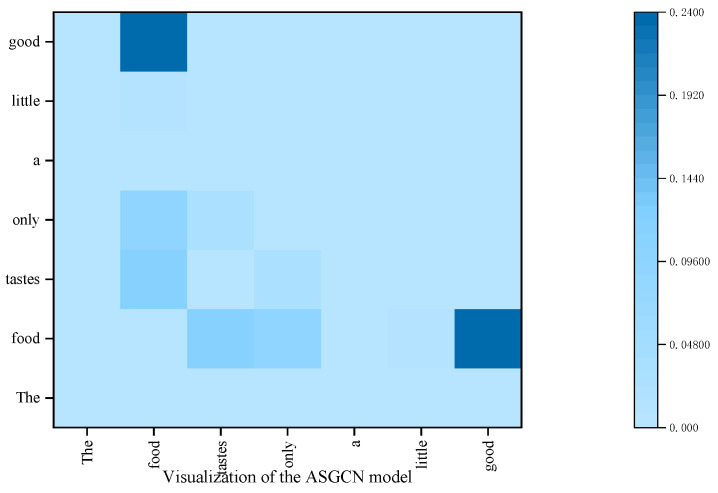
Attention weights of the ASGCN model.

To better illustrate that the proposed SDTGCN model enhances the performance of aspect sentiment analysis, we visualize the attention focus in different models for the sentence “*The food tastes only a little good*” in Figure 12 and Figure 13. The SDTGCN model utilizes a structured dependency tree to explore key opinion words related to the aspect from multiple perspectives. From the graph, it can be observed that the SDTGCN model assigns higher attention to the aspect “*food*” and the words“*only*”, “*little*”, “*good*”. Through comprehensive analysis, the model determines the sentiment polarity of the aspect as negative. This demonstrates that the SDTGCN model not only focuses on explicit opinion words, but also extracts crucial keywords important for determining the sentiment of the aspect.

In contrast, in the ASGCN model, higher attention is given to the aspect word “*food*” and “*good*”, while “*only*” and “*little*” receive lower attention. As a result, the model incorrectly analyzes the sentiment polarity of the aspect word ‘food’ as positive. Therefore, the proposed SDTGCN model not only concentrates on explicit opinion words, but also accurately explores the sentiment analysis of the aspect through structured dependency tree-based comprehensive analysis.

## 5. Conclusions

In this paper, we introduce a Structured Dependency Tree-based Graph Convolution Network (SDTGCN) for fine-grained aspect sentiment analysis. The SDTGCN model enriches the general dependency tree using positional information, sentiment common sense knowledge, part-of-speech tags, and syntactic dependency distances. This approach assigns higher weights to pivotal words relevant to aspects and lower weights to words unrelated to aspects, thereby enhancing the representation of aspects in relation to pivotal opinion words. Moreover, it considers the relationship between pivotal opinion words that are not directly dependent on each other through part-of-speech and syntactic dependency distance, reducing the distance between aspects and pivotal opinion words and thus improving sentiment classification performance. Finally, the SDTGCN model leverages a graph convolutional network with aggregated edge weights to sequentially aggregate information from enhanced neighbor nodes and subadjacent nodes based on node importance. This allows for the precise representation of specific aspects and enhances the performance of aspect-based sentiment analysis. Experimental results demonstrate the efficacy of our proposed model on five public datasets, outperforming other baseline models.

While this study achieved favorable results, there are still some limitations. First, the dataset used in this study is relatively small. Second, the SDTGCN model relies on the syntactic structure of sentences, and its effectiveness may be less pronounced for sentences lacking clear syntactic structures. It is constrained to datasets where the syntactic structure features are prominent or accurate dependency parsing is available. Thus, the proposed SDTGCN model may not perform well in datasets with less obvious syntactic structures or when dependency parsing accuracy is compromised. Therefore, in future work, we plan to enhance the semantic information of sentences to fully capture the emotional dependencies between specific aspects and contextual information. This involves addressing the limitations associated with syntactic structures and attempting to train the model on larger datasets.

## Figures and Tables

**Table 1:** Summary of the related work.

Models	RNN	Attention	CNN	GCN	GAT	Syntactic	Knowledge
TD-LSTM	✓						
ATAE-LSTM	✓	✓					
ASCNN	✓		✓				
R-GAT	✓	✓			✓	✓	
SK-GCN	✓	✓		✓		✓	✓
CDT	✓			✓		✓	
ASGCN	✓	✓		✓		✓	
RMN	✓	✓		✓		✓	
InterGCN	✓	✓		✓		✓	
GL-GCN	✓	✓		✓		✓	
SenticGCN	✓	✓		✓		✓	

**Table 2:** All notations used to explain the proposed model.

Notation	Type	Definition
*s*	Set	A sentence with n-words
$w_{i}$	One-hot vector	The i-th word in the sentence
$a_{i}$	One-hot vector	The i-th word in the aspect terms
n	Scalar	The length of the context word
m	Scalar	The length of the aspect word
$d_{w}$	Scalar	The dimension of word embedding
$d_{h}$	Scalar	The dimension of hidden representation
X	Matrix	The GloVe embedding matrix
D	Matrix	The adjacency matrix
P	Matrix	The position weight matrix
$s_{a}$	Set	The set of aspects in the sentence
E	Matrix	The sentiment knowledge matrix
A	Matrix	The aspect matrix
$W_{adj}$	Matrix	The adjacency enhanced dependency weight matrix
$W_{sub}$	Matrix	The subadjacency matrix
$H^{c}$	Matrix	HcThe final representation of the sentence

**Table 3:** Datasets.

Dataset	Positive		Neural		Negative	
	**Train**	**Test**	**Train**	**Test**	**Train**	**Test**
Lap14	994	341	464	169	870	128
Rest14	2164	728	637	196	807	193
Rest15	978	326	36	34	307	182
Rest16	1230	440	62	28	417	107
Twitter	1561	173	3127	346	1560	1743

**Table 4:** Comparison with the baseline models on five public datasets. Acc represents accuracy, F1 represents Macro-F1 score. Best results are in bold face and second best underlined.

Models	Lap14		Rest14		Rest15		Rest16		Twitter	
	**Acc**	**F1**	**Acc**	**F1**	**Acc**	**F1**	**Acc**	**F1**	**Acc**	**F1**
TD-LSTM	71.83	68.43	78.00	66.73	76.39	58.70	82.16	54.21	70.80	69.00
ATAE-LSTM	68.70	63.93	77.20	67.02	78.48	60.53	83.77	61.71	-	-
IAN	72.05	67.38	79.26	71.94	78.54	57.26	84.74	62.29	72.50	70.81
MemNet	70.64	65.17	79.61	69.64	77.31	58.28	85.44	65.99	71.48	69.90
AOA	72.62	67.52	79.97	70.42	78.17	57.02	87.50	66.21	72.30	70.20
TNet-LF	74.61	70.14	80.42	71.03	78.47	59.47	89.07	70.43	72.98	71.43
ASCNN	72.62	66.72	81.73	73.10	78.47	58.90	87.39	64.56	71.05	69.45
R-GAT	77.42	73.76	83.30	76.08	80.83	64.17	88.92	70.89	75.57	73.82
SK-GCN	73.20	69.18	80.36	70.43	80.12	60.70	85.17	68.08	71.97	70.22
CDT	77.19	72.99	82.30	74.02	-	-	85.58	69.93	74.66	73.66
ASGCN	75.55	71.05	80.77	72.02	79.89	61.89	88.99	67.48	72.15	70.40
BiGCN	74.59	71.84	81.97	73.48	81.16	64.79	88.96	70.84	74.16	73.35
AGCN	75.07	70.96	80.02	71.02	80.07	62.70	87.98	65.78	73.98	72.48
RMN	74.50	69.79	81.16	73.17	80.69	64.41	88.75	71.54	-	-
InterGCN	77.86	74.32	82.23	74.01	81.76	65.67	89.77	73.05	-	-
GL-GCN	76.91	72.76	82.11	73.46	80.81	64.99	88.47	69.64	73.26	71.26
SenticGCN	77.90	74.71	**84.03**	75.38	82.84	67.32	90.88	75.91	-	-
**SDTGCN**	**78.64**	**75.50**	83.82	**76.13**	**83.21**	**67.07**	**91.53**	**77.08**	**76.25**	**74.59**

**Table 5:** Case study. We use different models to analyze various cases, where “P”, and “N” represent positive, and negative sentiments, respectively. Red and blue are used to emphasize positive and negative emotions, while yellow is used to highlight aspects.

Case	IAN	ASGCN	SDTGCN	Ground Truth
The screen on this phone is not very good.	P	N	N	N
The food tastes only a little good.	N	P	N	N
The meat in this good restaurant is stale.	N	N	P	P

## Data Availability

Data are contained within the article.
